# 

**Published:** 1897-10

**Authors:** 


					﻿CONTENTS.
ORIGINAL ARTICLES:
Traumatic (?) Stricture of the Rectum ....................  477
1. Calcareous Uterine Fibroid. 2. Appendicitis ............ 484
A Clinical Report on Gude’s Pepto-Mangan................... 487
SELECTIONS AND ABSTRACTS:
Gunshot Wounds and Their Treatment......................... 489
The Ether-Chloroform Controversy........................... 494
Indifference of Patients.................................   497
Value to the Public of State Medical Societies.............. 499
The immediate Reduction of the Angular Deformity of Spinal
Caries ...................................... ...........	506
Fracture of the Penis....................................... 508
A New Method of Inviting sleep ............................. 509
Examination of Throats of Children ......................... 511
SELECTIONS AND ABSTRACTS:
The Differential Diagnosis Between Malarial and Typhoid Fevers... 512
Ninth Annual Meeting of the Tii-State Medical Society.. 514
Common Sense in the use of Antiseptics...........       517
Ingrown Toe Nails....................................   518
That Wave of Prosperity.............................    519
EDITORIAL:
Atlanta anc* the Yellow Jack..........................  520
Abstract of Letter From Dr. Sajous to Dr. Gaston ..•... 522
BUSINESS DEPARTMENT.
Special Notices................................ 523,525,	526
PRESCRIPTION DEPARTMENT.
Prescriptions........... ,. ........................... 529
				

